# Relationship of smartphone use severity with sleep quality in bipolar patients

**DOI:** 10.1192/j.eurpsy.2023.472

**Published:** 2023-07-19

**Authors:** J. Kim, S.-H. Kim

**Affiliations:** Department of Psychiatry, Korea University Guro Hospital, Seoul, Korea, Republic Of

## Abstract

**Introduction:**

Maintaining a good sleep-wake cycle is an important factor for the prognosis and management of bipolar disorder. However, studies on the to various technological advances including smartphoe usage affecting inter-episodic sleep quality are yet relatively less thoroughly investigated.

**Objectives:**

This study aims to identify the association between smartphone usage and inter-episodic sleep quality of bipolar patients.

**Methods:**

A total 52 Bipolar I or II subjects who were euthymic for at lest 6 months were included in this analysis. Pearson correlation analysis was used to examine the association among psychological assessments, including the Pittsburgh Sleep Quality Index (PSQI-K), Smartphone Addiction Scale (SAS), Hamilton Depression Rating Scale (K-HDRS), Young Mania Rating Scale (K-YMRS), and Multidimensional Scale of Perceived Social Support (MDPSS). Significant results were then analyzed using a multiple linear regression analysis with PSQI-K as the dependent variable to assess the impact of clinical variables on sleep quality.

**Results:**

PSQI-K was positively correlated with SAS (r = 0.457, p < 0.001), K-HDRS (r = 0.447, p < 0.001), and negatively correlated with MDPSS (r = -0.336, p < 0.05). Smartphone use, depressive symptoms, and perceived social support seemed to explain 35.7% of sleep quality. After adjusting for confounders, more smartphone use and more severe depressive symptoms were associated with poor sleep quality (SAS: β = 0.383, p = 0.002; K-HDRS: β = 0.339, p = 0.006), but perceived social support did not reach statistical significance (MDPSS: β = -0.204, p = 0.086).

**Conclusions:**

The results of this study show that the more a person uses a smartphone, the worse their sleep is. This effect is significant, even when other factors are taken into account. These results support the possibility that improving the degree of smartphone use could be an essential intervention target for improving sleep quality during the inter-episode period in patients with bipolar disorder.

**Disclosure of Interest:**

None Declared

